# Topical Steroid-Induced Perioral Dermatitis (TOP STRIPED): Case Report of a Man Who Developed Topical Steroid-Induced Rosacea-Like Dermatitis (TOP SIDE RED)

**DOI:** 10.7759/cureus.14443

**Published:** 2021-04-12

**Authors:** Kyra L Diehl, Philip R Cohen

**Affiliations:** 1 Osteopathic Medicine, Western University of Health Sciences, Pomona, USA; 2 Dermatology, San Diego Family Dermatology, National City, USA

**Keywords:** corticosteroid, rosacea, topical, calcineurin, clindamycin, dermatitis, doxycycline, perioral, steroid, tetracycline

## Abstract

The long-term use of topical corticosteroids can result in rosacea-like dermatitis or facial perioral dermatitis. The case of a 54-year-old man is described who developed topical corticosteroid-induced perioral dermatitis (TOP STRIPED), and the features of topical corticosteroid-induced rosacea-like dermatitis are reviewed. The man presented with a painful erythematous facial eruption. Additional history revealed that he had been applying a high-potency topical corticosteroid twice daily to the affected area. Correlation of the clinical history and cutaneous examination established a diagnosis of topical corticosteroid-induced rosacea-like dermatitis (TOP SIDE RED). Treatment of the patient’s TOP SIDE RED included not only discontinuing the high-potency corticosteroid but also initiating topical and oral antibiotics. In addition, a low-potency topical corticosteroid and metronidazole gel were also applied to the affected area. His facial rash resolved within three months and has not recurred. TOP STRIPED, also referred to as TOP SIDE RED, is an adverse side effect associated with the use of high-potency topical corticosteroids to the face. Management includes discontinuing the corticosteroid. Additional treatment may include a low-potency topical corticosteroid, antibiotics (systemic or topical or both), and/or topical calcineurin inhibitors, such as tacrolimus or pimecrolimus.

## Introduction

Corticosteroids are used for the treatment of multiple systemic and cutaneous conditions. The route of administration may be intravenous, oral, intermuscular, intralesional, or topical. Topical corticosteroids vary in potency [1,2].

Perioral dermatitis and rosacea are facial conditions. Some of the skin lesions of those conditions have similar morphology and distribution. There are several postulated mechanisms for the development of perioral dermatitis and rosacea; however, prolonged duration of application of high-potency topical corticosteroids can result in the appearance of a dermatitis that has been referred to as either topical steroid-induced perioral dermatitis (TOP STRIPED) or topical steroid-induced rosacea-like dermatitis (TOP SIDE RED) [3-5].

A man with TOP SIDE RED is described. His facial condition occurred after he applied a high-potency corticosteroid cream, which had been prescribed for his hand dermatitis, to his face daily for nearly two months. His dermatosis eventually resolved after he was treated with not only oral and topical antibiotics, but also a lower-potency topical corticosteroid cream. The features of TOP STRIPED are reviewed.

## Case presentation

A 54-year-old man presented for evaluation of a painful and persistent red rash on his face. His past cutaneous history was significant for hand dermatitis of several years. He had previously seen an allergist, and testing revealed sensitivity to dust mites and cockroaches. He also had thin-layer rapid-use epicutaneous patch testing to 30 allergens; all of these were negative for contact allergy. 

His hand dermatitis was treated topically with clobetasol propionate 0.05% cream. After six months, his dermatitis eventually cleared without recurrence. Follow-up 1.5 years later showed continued resolution of his hand dermatitis. 

**Figure 1:**
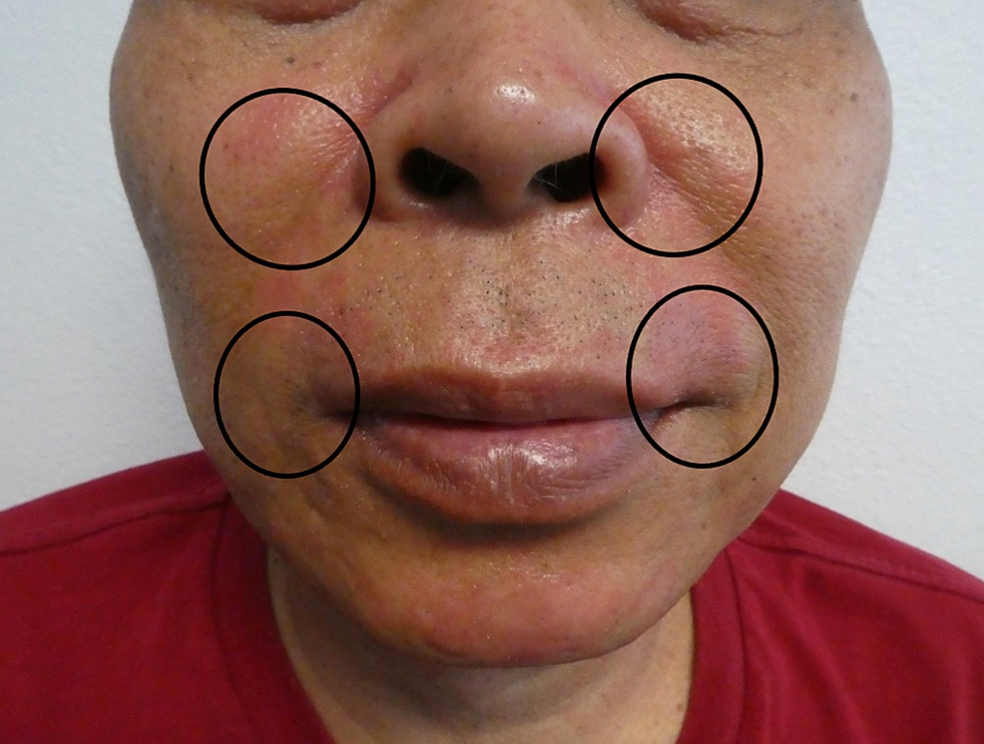
Clinical presentation of TOP STRIPED A 54-year-old man presenting with tender erythema on his face after applying clobetasol 0.05% cream for two months. The clinical features of his TOP STRIPED are shown (black ovals) on the frontal facial view. TOP STRIPED, topical steroid-induced perioral dermatitis

**Figure 2:**
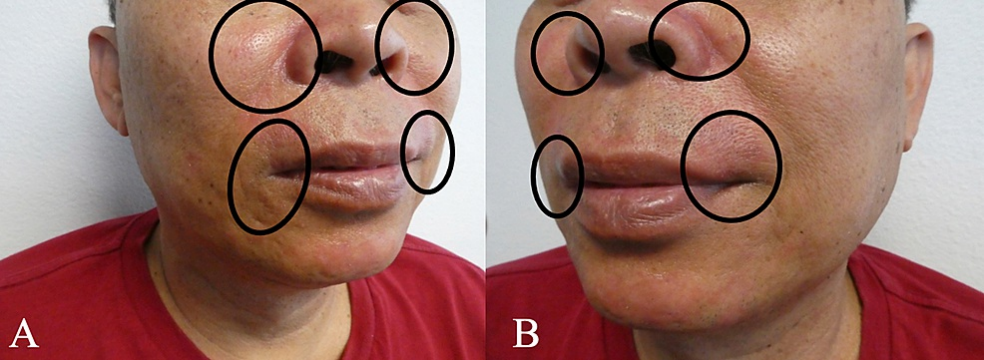
TOP SIDE RED presents as painful facial erythema Right side (A) and left side (B) facial views of TOP SIDE RED show malar, perinasal, and perioral erythema (black ovals) TOP SIDE RED, topical steroid-induced rosacea-like dermatitis

At his follow-up visit 18 months after the initial clearing of his hand dermatitis, he presented with perinasal, perioral, and malar tender erythema on his face (Figure 1 and Figure 2). During the prior two months, he had been applying the clobetasol cream once or twice daily on his face. The areas of application had been dry and were in a distribution typically affected by seborrheic dermatitis. 

Correlation of his clinical history and morphologic lesion appearance established a diagnosis of TOP SIDE RED. Initial treatment included discontinuing the clobetasol cream; a low-potency corticosteroid cream (hydrocortisone acetate 2.5% cream) was initiated. The patient initially declined oral antibiotic therapy; therefore, topical clindamycin 1% solution twice daily was prescribed.

At the subsequent visit, two months later, the dermatitis on his face had persisted. He concurred with starting an oral antibiotic, and doxycycline monohydrate, 100 mg twice daily, was begun. He also continued using topical clindamycin 1% solution twice daily and hydrocortisone 2.5% cream once daily. 

**Figure 3:**
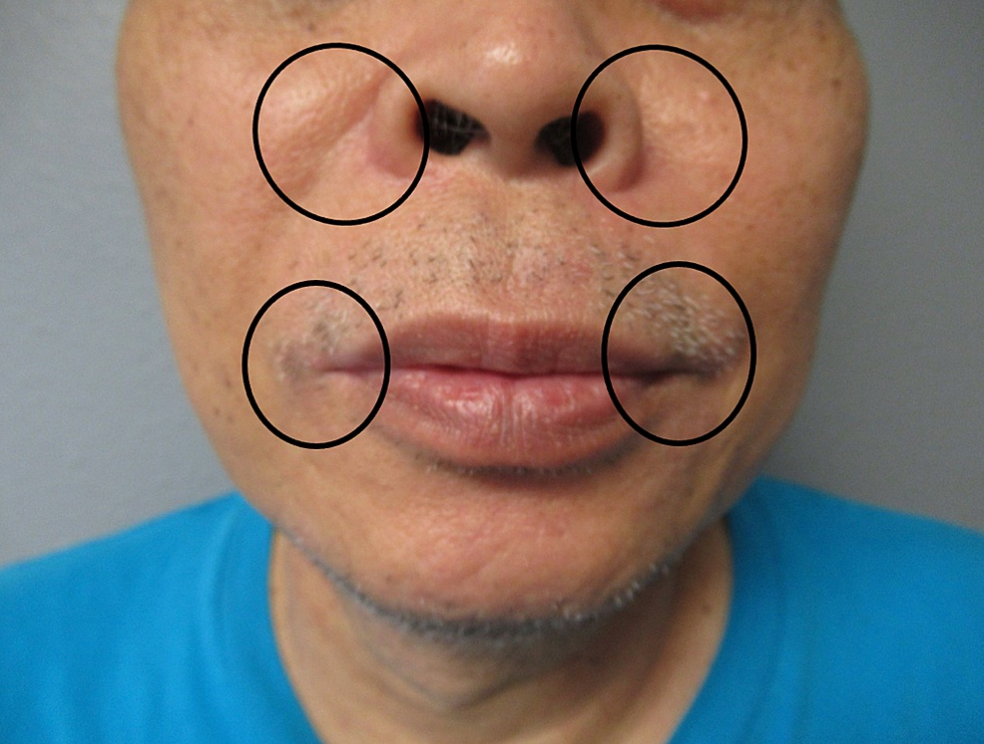
Resolution of TOP SIDE RED Three months after treatment with topical and oral antibiotics, and topical hydrocortisone acetate 2.5% cream, the patient achieved remission without recurrence of TOP SIDE RED. The previously affected malar, perinasal, and perioral areas of erythema (within the black ovals) are now resolved TOP SIDE RED, topical steroid-induced rosacea-like dermatitis

**Figure 4:**
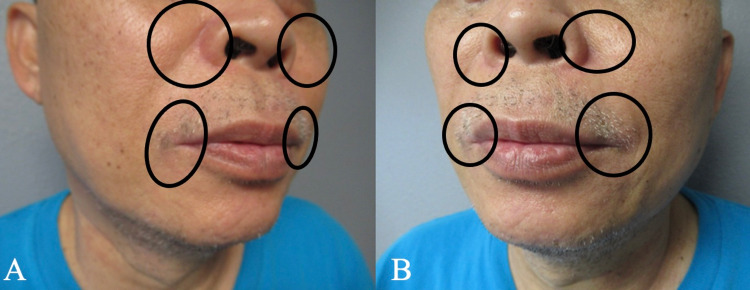
TOP STRIPED resolved after treatment with oral doxycycline monohydrate, topical clindamycin 1% solution, and topical hydrocortisone acetate 2.5% cream Right side (A) and left side (B) views of resolved TOP STRIPED. The previously affected red areas (shown within black ovals) are now flesh-colored TOP STRIPED, topical steroid-induced perioral dermatitis

When he returned, after four weeks, his face was clear (Figure 3 and Figure 4). The clindamycin solution irritated his nostrils and was stopped. His management now included tapering the oral antibiotic (to once daily for two weeks and then once daily Monday, Wednesday, and Friday for two weeks), adding metronidazole 0.75% gel twice daily and continuing the hydrocortisone cream once daily. 

At his next visit, again after four weeks, his perioral dermatitis remained resolved. He was able to stop both the metronidazole gel and the hydrocortisone cream. At the subsequent follow-up, two months later, his hands still remained clear and there was no recurrence of the facial dermatitis. 

## Discussion

Topical corticosteroids can be used for the treatment of papulosquamous disorders, such as dermatitis, including seborrheic dermatitis. They have a wide range of potencies. They range from ultra-potent Class I formulations such as clobetasol propionate 0.05%, which may be used to treat hand dermatitis, to lower-potency Class VII products, such as hydrocortisone acetate 0.5%, which may be used to manage facial seborrheic dermatitis [1,2]. 

There are adverse effects that may be associated with topical corticosteroids. These include atrophic changes (such as purpura, striae, and telangiectasias) and infection (such as tinea incognito). In addition, an adverse effect of topical corticosteroids--similar to our patient--includes perioral dermatitis or rosacea-like dermatitis [1-3]. 

The first case of TOP SIDE RED was reported in 1957 by Frumess and Lewis, who described it as a dermatosis similar to seborrheic dermatitis. They referred to the condition as “light-sensitive seborrheid” [4]. Subsequently, several terms have been used to describe this condition (Table 1) [4-10]. 

**Table 1:** Nomenclature of facial dermatitis associated with topical corticosteroids CR, current report ^ a^The authors have suggested the acronym, SIRD, for steroid-induced rosacea-like dermatitis ^b^The authors suggest the acronym, TOP STRIPED, for topical steroid-induced perioral dermatitis ^c^The authors suggest the acronym, TOP SIDE RED, for topical steroid-induced rosacea-like dermatitis

Nomenclature	Reference
Corticosteroid-induced acne rosacea	[5]
Corticosteroid-induced perioral dermatitis	[5]
Light-sensitive seborrheid	[7]
Perioral dermatitis	[8]
Rosacea-like dermatitis	[6,9]
Rosacea-like eruption from topical steroid	[4]
Steroid dermatitis resembling rosacea	[4]
Steroid rosacea	[10]
Steroid-induced rosacea-like dermatitis^a^	[4]
Topical steroid-induced perioral dermatitis^b^	CR
Topical steroid-induced rosacea-like dermatitis^c^	CR

The exact incidence of TOP SIDE RED remains to be determined. However, the condition most frequently occurs in patients between 40 and 50 years of age and is more common in women than men [2-4]. 

The pathogenesis of TOP SIDE RED is associated with repeated application of topical corticosteroids to the face. When more potent corticosteroids are applied, the condition may occur after a shorter duration of application. In contrast, when lower-potency corticosteroids are repeatedly applied to the face, a longer duration of application may be required. Often, when the individual stops applying the topical corticosteroid, the dermatitis flares and the person requires an increasingly higher-potency topical corticosteroid to temporarily resolve their skin problem [1,2]. 

There are several potential sources for the topical corticosteroid that a patient is applying to his/her face. Similar to our patient, they may have previously been prescribed a high-potency topical corticosteroid for a skin problem affecting another area of their body and then decided, on their own, to apply the same medication to their face. In other circumstances, they may have obtained the medication from a family member or a friend. 

In addition, the higher-potency class I topical corticosteroids are formulated in lower percentages, whereas the lower-potency Class VII topical corticosteroids are often formulated in higher percentages. For example, the Class I clobetasol is prepared as a 0.05% product in contrast to the Class VII hydrocortisone, which can be prescribed as a 2.5% agent. The patient may incorrectly be attempting to apply a less strong preparation based on the percentage that they view on the tube of medication and are, therefore, inadvertently applying a higher-potency agent to their face [1,2]. 

The diagnosis of TOP SIDE RED may be suspected clinically. However, it is usually confirmed based upon the history of medication use obtained from the patient. Furthermore, the clinician may need to specifically inquire about topical facial medications because the individual may not realize that the medicine they are applying to their face is causing the skin condition [5].

There are several conditions in the clinical differential diagnoses of TOP SIDE RED (Table 2) [3-10]. They include not only acneiform conditions, such as rosacea, perioral dermatitis, and acne vulgaris, but also other skin conditions, such as dermatitis, seborrheic dermatitis, and tinea facei. They also include systemic conditions, such as dermatomyositis, lupus erythematosus, and sarcoidosis [3-10].

**Table 2:** Clinical differential diagnosis of topical corticosteroid-associated facial dermatitis

Conditions	References
Acne vulgaris	[4,6-10]
Cutaneous lupus erythematous	[4-10]
Dermatitis (acute)	[4,6-10]
Dermatomyositis	[4,6-10]
Lupus miliaris disseminatus facei	[4,6-10]
Perioral dermatitis	[4,6-10]
Polymorphous light eruption	[4-10]
Rosacea	[4,6-10]
Sarcoidosis	[4,6-10]
Seborrheic dermatitis	[4,6-10]
Steroid acne	[4,6-10]
Systemic lupus erythematosus	[4-10]
Tinea facei	[4-10]

Two acronyms may aid in recalling the pathogenesis and clinical features of this condition. The acronym TOP SIDE RED refers to topical steroid-induced rosacea-like dermatitis. TOP are the first three letters of ‘topical.’ S is the first letter of ‘steroid.’ IDE are the first, third, and sixth letters of ‘induced.’ RE are the first and sixth letters of ‘rosacea.’ D is the first letter of ‘dermatitis.’ The acronym TOP STRIPED refers to topical steroid-induced perioral dermatitis. TOP are the first three letters of ‘topical.’ ST are the first two letters of ‘steroid’; R is the fourth letter of ‘steroid.’ I is the first letter of ‘induced.’ PE are the first two letters of ‘perioral.’ D is the first letter of ‘dermatitis.’ 

A skin biopsy is usually not required to establish the diagnosis of TOP SIDE RED. However, it may be performed to exclude other conditions in the clinical differential diagnosis. When performed, microscopic examination of the tissue specimen shows not only eczematous changes of the epidermis (such as acanthosis and overlying parakeratosis) and dermis (such as edema) but also connective tissue hypertrophy and sebaceous hypoplasia. In addition, neutrophil-containing follicular abscesses and noncaseating granulomas may be present in the dermis [4].

Epidermal thinning may occur within weeks of using the corticosteroid. Prolonged use of topical corticosteroids will also result in inhibition of collagen synthesis, leading to diminished dermal volume. However, the epidermis may begin to return to normal within two months after stopping the application of the corticosteroid [1,2,4].

The main treatment of TOP SIDE RED is discontinuing the application of the high-potency corticosteroid. Some of the earlier investigators advocated that no additional topical therapy be initiated. However, they observed that the weeks following discontinuation of the high-potency corticosteroid were very difficult for the patient to tolerate. Therefore, other researchers have recommended progressive tapering of the potency of the topical corticosteroid. 

In addition, other interventions have also been suggested [1,3,4]. These include the initiation of systemic and topical antibiotics. The effectiveness of the antibiotics may be associated with the anti-inflammatory effects of the drug [3]. 

Tetracyclines are often the first option as a systemic antibiotic. In addition to tetracycline, these may include doxycycline or minocycline. In patients who cannot tolerate tetracycline, oral metronidazole has been used. 

Topical antibiotics can also be initiated. For example, clindamycin 1% or erythromycin 2% or metronidazole 0.75% or 1.0% may be applied to the affected area once or twice daily. A lotion base may be optimal; however, solution, gel, and cream preparations may also be used [1,3,5]. 

More recently, some investigators have advocated the use of topical calcineurin inhibitors. These include topical pimecrolimus 1% cream or tacrolimus 0.03% or 0.1% ointment. These treatments have been shown to provide quicker initial improvement and overall resolution of TOP SIDE RED [3,4].

Our patient initially declined systemic antibiotic therapy. Sustained resolution was eventually achieved once systemic antibiotic therapy, along with topical antibiotics and tapering of topical corticosteroid, was initiated. He has subsequently remained without recurrence of his TOP SIDE RED.

## Conclusions

Topical corticosteroids may be associated with adverse cutaneous events, including TOP SIDE RED, which has also been referred to as TOP STRIPED. The facial dermatitis results from high-potency topical corticosteroids being applied to the face and presents as painful erythema, which can be not only perioral and perinasal, but also affect the cheek area and mimic rosacea. Once the diagnosis is established, the main therapeutic intervention is discontinuing the high-potency corticosteroid. In addition, tapering the topical corticosteroid, antibiotics (systemic and/or topical), and calcineurin inhibitors may be efficacious, adjuvant therapies.
